# Spatiotemporal models with confounding effects: application on under-five mortality across four sub-Saharan African countries

**DOI:** 10.3389/fpubh.2025.1408680

**Published:** 2025-01-23

**Authors:** Haile Mekonnen Fenta, Ding-Geng Chen, Temesgen T. Zewotir, Najmeh Nakhaei Rad

**Affiliations:** ^1^Department of Statistics, University of Pretoria, Pretoria, South Africa; ^2^Department of Statistics, Bahir Dar University, Bahir Dar, Ethiopia; ^3^College of Health Solutions, Arizona State University, Phoenix, AZ, United States; ^4^School of Mathematics, Statistics and Computer Science, College of Agriculture Engineering and Science, University of KwaZulu-Natal, Durban, South Africa

**Keywords:** spatial random effects, confounding, spatiotemporal models, space-time interactions, variance partitioning

## Abstract

**Background:**

Different strategies have been developed to minimize under-five mortality (U5M) in sub-Saharan African (sSA) countries; however, it is still a major health concern for children in the region. Spatiotemporal modeling is important for areal data collected over time. However, when the number of time points and spatial areas is large and the areas are disconnected, fitting the model becomes computationally complex because of the high number of required parameters to be estimated. Therefore, the main aim of this study is to adopt a spatiotemporal dynamic model that includes the confounding effects between time, space, and their interactions with fixed covariates, with a special emphasis on U5M across disconnected sSA countries.

**Method:**

We used nationally publicly representative Demographic and Health Survey (DHS) data for the period from 2000 to 2020. Bayesian spatiotemporal hierarchical modeling with an integrated nested Laplace approximation (INLA) program was used to model the spatiotemporal distribution of U5M among children across 37 districts located in four disconnected sSA regions: Ethiopia, Nigeria, Zimbabwe, and Ghana.

**Results:**

A total of 170,356 under-five children from 37 districts were considered, and 15,467 died before the age of five. The relative risk of U5M in the first DHS was 2.02, which sharply decreased to 0.5 in the recent phase. The proportion of improved access to water, sanitation, clean fuel use, urbanization, and access to health facilities in the district had a significant negative association with U5M. The higher the proportion of these covariates, the lower is the prevalence of childhood mortality.

**Conclusion:**

This study revealed evidence of strong spatial, temporal, and interaction effects that influence under-five mortality risk across districts. Improving the women’s literacy index, access to improved water, the use of clean fuel, and the wealth index are associated with an improvement in the risk of mortality among under-five children across the districts. Districts in Nigeria and Ethiopia have the highest risk of U5M; hence, districts in these countries require special attention.

## Introduction

Under-five mortality (U5M) is the probability that a certain child will die before celebrating the age of five. This is considered one of the benchmarks of a given country’s health indicators and progress toward the achievement of development goals (1, 2). In 1990, the World Summit for Children set the goal of reducing U5M births by a third (70 per 1,000 live) between 1990 and 2000 (3). This was approved by the Millennium Development Goal (MDG) 4, which proposed a two-thirds reduction between 1990 and 2015 (4). As part of the Sustainable Development Goals (SDG), target 3.2 seeks to end preventable under-five child deaths and reduce the U5M rate to 25 per 1,000 live births by 2030 (5). Between 1990 and 2015, a 53% reduction in U5M was obtained globally (1, 6), but the lowest reduction was recorded in sub-Saharan African (sSA) countries. The international development goal benchmark on the U5M has improved over time owing to the accessibility of data and increased advanced methodology. In all countries, the burden of U5M is expected to be concentrated in some districts over time; identifying these highly concentrated areas and directing suitable interventions to these areas will accelerate national U5M reductions to ensure effective resource allocation.

The spatiotemporal models have become increasingly common in different areas of applications, such as public health and medicine, epidemiology, energy research, environmental sciences (7, 8), etc. To describe the pattern of disease counts and identify the hot/cold spot areas of the incidence level as well as time trends, spatiotemporal disease mapping models are commonly employed (9, 10). Moreover, in spatial and spatiotemporal statistics, the spatial dependency between neighboring regions should be properly defined prior to the analysis. The intrinsic conditional autoregressive (ICAR) model is a widely used approach for specifying spatial dependency (9). In the ICAR model, the map comprises nodes and edges that represent the respective areas and their neighboring relationships (11). In connected maps, all the prior nodes are connected, but sometimes a disconnected graph can arise when there are edges and nodes with no neighbors or when the study areas are split with subgraphs (11). There is limited literature on the specification of the ICAR approach for disconnected graphs, and few researchers have highlighted the situation of ICAR in disconnected regions (11, 12). We employed these techniques using childhood under-five mortality in four sSA-disconnected countries (Ethiopia, Ghana, Nigeria, and Zimbabwe). Bayesian framework approaches have been used, including parametric and non-parametric (13, 14) formulations of spatial areas, time trends, and space–time interactions. The parameter estimates were obtained from Markov chain Monte Carlo (MCMC) algorithms, which are computationally expensive and might also induce Monte Carlo error in parameter estimates, especially for space–time interaction effects (15, 16). As a result, to compute the posterior marginals of all parameters of interest, a new approach called the integrated nested Laplace approximation (INLA) was developed (17), which has a short computational time and is also more effective (18). Many studies on the spatial and temporal distribution of U5M have been conducted (1, 3, 19–22); however, their conclusions were limited because they failed to account for spatial, temporal, and spatiotemporal random effects with covariates. Often, in disease mapping, we assume that the graph is connected, meaning that all nodes connect to at least one other node. However, disconnected graphs can arise when there is free space between regions (islands with no neighbors or when the study region is split), resulting in separate subgraphs. Another important aspect is that using INLA for disconnected regions has a lower error than using separated regions (12, 23). We also aimed to find an overall estimate of the under-five mortality across the four sSA countries. This is because, in the spatiotemporal context, the covariates can exhibit temporal patterns in each area and spatial patterns in each year. As the model includes both spatial and temporal random effects, along with interaction terms, the source of confounding covariates may be temporal, spatial, or their interaction. Therefore, in this study, we adopt a model that accounts for spatiotemporal models with covariates and temporal, spatial, and spatiotemporal random effects to estimate the U5M rate across the districts of four disconnected countries in sSA.

## Data sources

This study used the nationally representative Demographic and Health Survey (DHS) dataset, mainly conducted in low-income countries^1^ and shapefiles of administrative districts.^2^ In the DHS, multistage sampling was used to select the sample for each survey from the countries included in this analysis. The first step of the sampling procedure involved the selection of clusters (enumeration areas, or EAs), followed by systematic household sampling within the selected EA. The number of clusters is the first stage, which is selected from the list of EAs created in the recent population census of each country and households that are randomly selected in each EAs. From the selected households, women aged 15–49 years were selected for in-depth interviews (24). The DHS questionnaire was revised over different phases: the first phase DHS (1997–2003), second phase (2004–2008), third phase (2009–2013), fourth phase (2014–2018), and the most recent DHS, eighth phase (2019–2023), to observe the differences in the spatial, temporal, and spatiotemporal prevalence and associated factors of the U5M rate across districts in the study areas.

**Figure 1:**
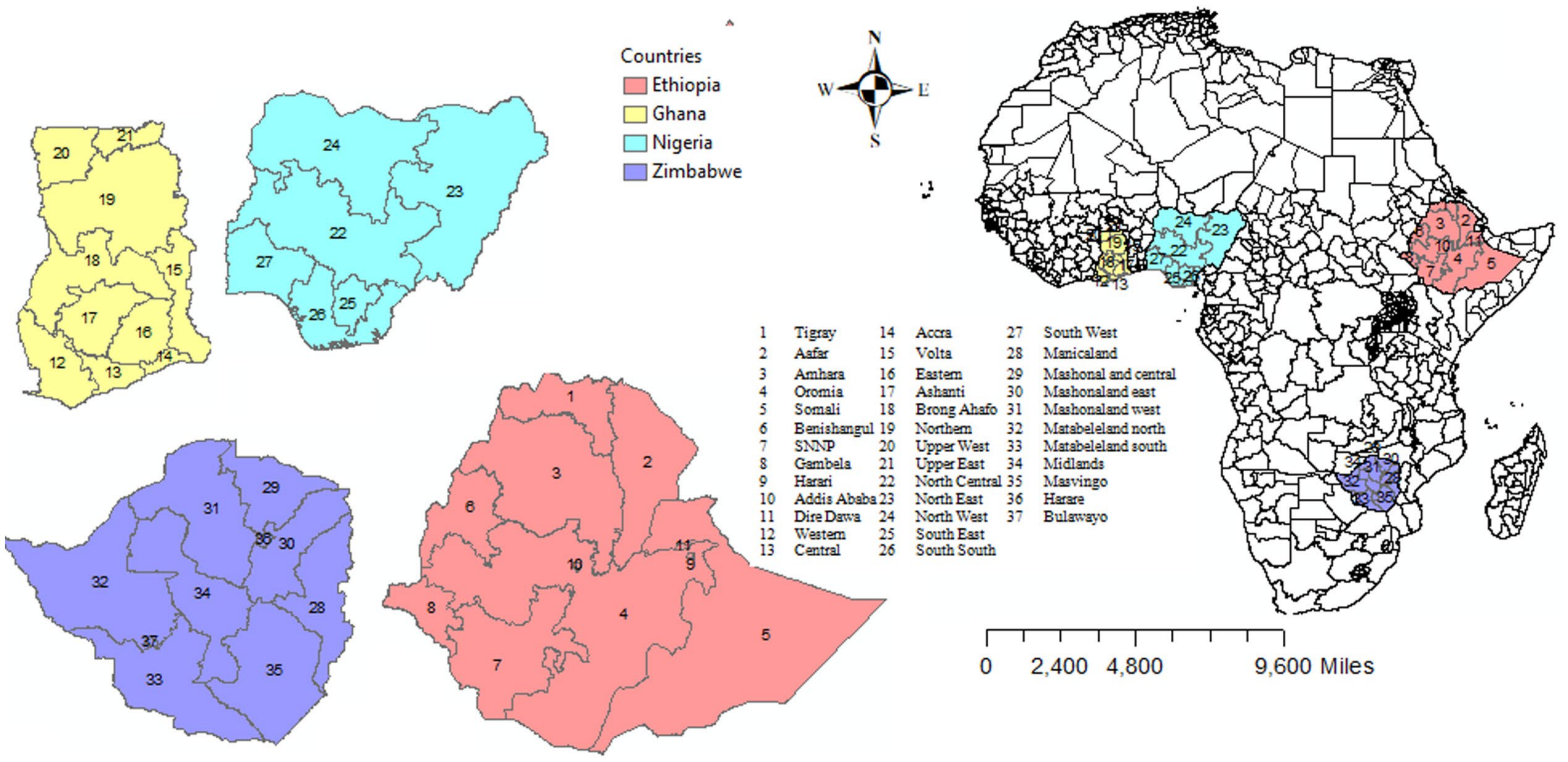
Map of eligible sub-Saharan African countries included in the study.

**Figure 2:**
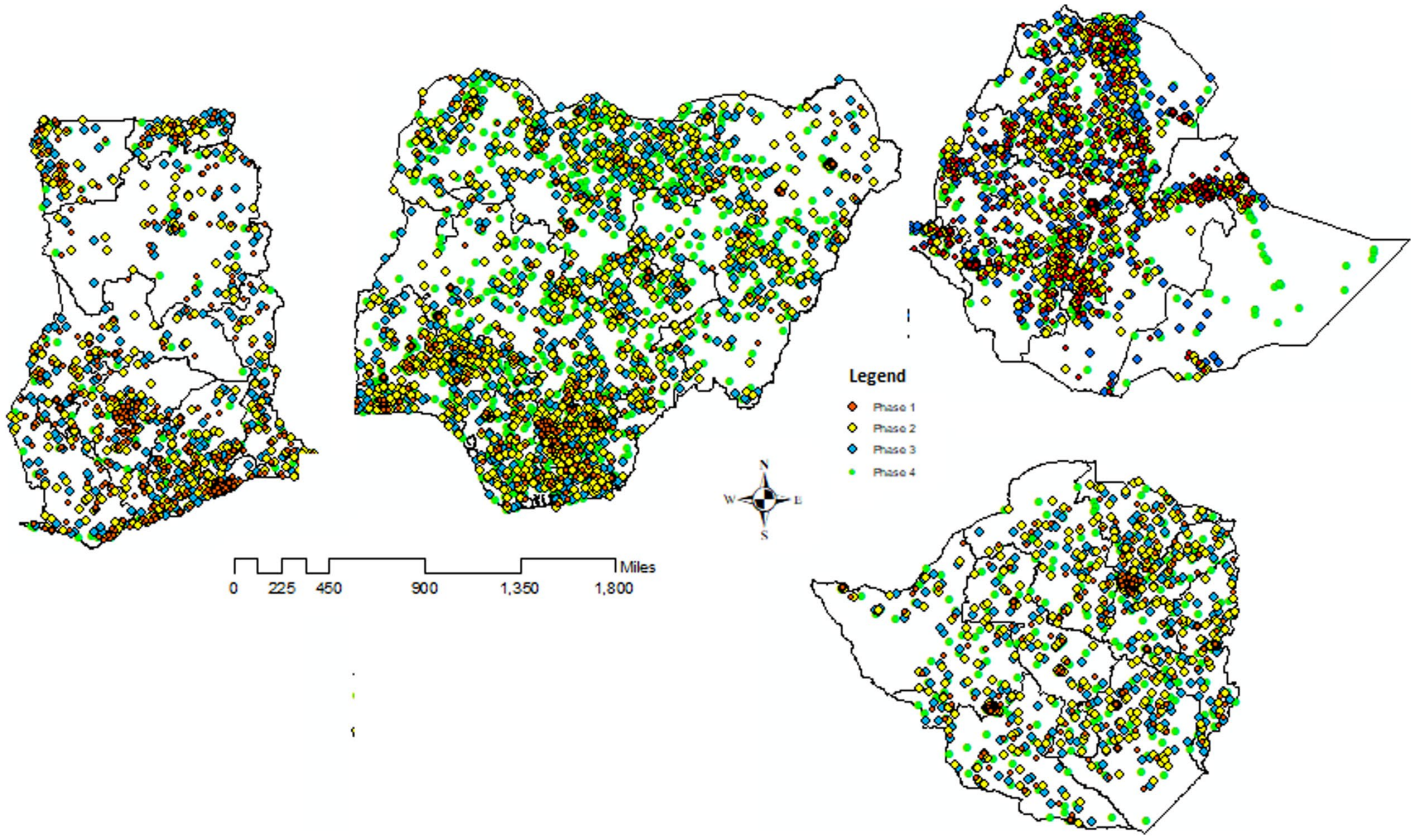
Locations of four disconnected countries with their clusters (Enumeration Areas) in the four phases of DHS that we consider, with boundaries of 37 districts in the four sSA countries.

The DHS contains the population size of each country/district, with different geospatial covariates. The DHS has two-stage designs, and the number of clusters is the first stage, which is selected from the list of enumeration areas (EAs) created in the recent population census of each country. Households were randomly selected in each EA. From the selected households, women aged 15–49 years were selected for in-depth interviews (24). The sSA is a portion of the African continent and consists of 49 countries, including Ethiopia, Ghana, Nigeria, and Zimbabwe (25). For this study, we retained the DHS data collected in sSA countries that had at least four surveys representative at a sub-national (district, region) level, with a consistent set of district boundaries across DHS surveys. This requirement is very important for pooling information from different surveys across time. Moreover, when district boundaries changed over time across the surveys, we used the location of clusters (GPS) to relocate them into coherent district units. This led us to select 16 surveys conducted in four disconnected sSA countries (Ethiopia, Nigeria, Ghana, and Zambia) between 2000 and 2020. Birth record files of under-five children from the sSA countries, which consisted of data on the full birth history of all reproductive women interviewed and information on health indicators of fertility and mortality rates, were used (Figure 1). Figure 2 shows the locations of 1,310, 2,230, 3,287, and 2,632 clusters in the four disconnected sSA countries over four different DHS data collection periods.

### Outcome variable

The outcome variable of interest for this study was the under-five mortality rate per 1,000 live births (19–21). The values of the variables were dichotomized as (1 = yes and 0 = no). The number of under-five child mortalities aggregated at the district level of the four disconnected sSA countries over the study period from 2000 to 2020 in the five preceding DHS survey rounds was the outcome of interest.

### Independent variables

The independent variables extracted were based on a review of the literature (19–22, 26–29). The independent variables were also aggregated over the districts, and the proportion of the variable of interest was computed. The variables included in the analysis are summarized in Table 1.

**Table 1:** The description of the covariates included in the model.

Child level covariates	Descriptions
% of children’s nourished (no CIAF)	The proportion of children with normal nutritional status
% female children	The proportion of female children
% of children with a dietary diversity score	The proportion of children with at least a minimum dietary diversity score
% of child born	The proportion of children of birth order
Maternal/household-level covariates	Description
% of women with literacy	The proportion of women with a literacy rate
% of mothers below the median age	The proportion of mothers below the median age
% of women with high autonomy	The proportion of women with low autonomy
% of access sanitation facilities	Percentage of population using at least basic sanitation services
% access to safe water	The proportion of households with improved water
% of clear fuels used	The proportion of households with use of clear fuel
% of women with media exposure	The proportion of women with media exposure
% of the working status of the mother	the proportion of women with working status
% of the working status of the father	The proportion of men with working status
% of households live in rural areas	The households living the rural areas
% of health facility	The percentage of women who delivered at health facilities
% of wealth quantile (WQ)	The proportion of households with a high poverty rate

CIAF, composite index for anthropometric failure (44) and dietary diversity (44, 45).

### Statistical models

Several statistical approaches to model spatiotemporal data features have been implemented in the last two decades within the disease mapping framework (15, 16), and have recently become extensively used to describe the temporal evolution of district patterns of rates. In majority of previous studies, the hierarchical Bayesian under the fully Bayes approach based on Markov Chain Carlo (MCMC) methods (9, 16, 30) was used to model the spatiotemporal dataset; however, a novel method called integrated nested Laplace approximation (INLA) has become an alternative estimation procedure, which overcomes some of the limitations of the MCMC estimation technique, such as the computation burden needed when high-dimensional datasets are available (17, 31, 32).

Let $A_{i}=\left\{i=1,\cdots ,S\right\}$ be the areas to be considered and $N_{i}=\left\{j\in A;i\in j\right\}$ the set of all areas and $T_{t}=\left\{t=1,\cdots ,T\right\}$ be the time considered in the study. Let $x_{\mathrm{it}}^{T}=\left(x_{\mathrm{it}1},\cdots ,x_{itk}\right)$ is the vector of standardized spatiotemporal covariates for fixed effects in areal units i and time t, $\beta ={\left(\beta_{1},\cdots ,\beta_{k}\right)}^{T}$ is the k -vector of the fixed effect parameters. Let $O_{\mathrm{it}}$ and $e_{\mathrm{it}}$ , respectively, be the observed and expected number of subjects in area i at period t, $N_{\mathrm{it}}$ is the total population in area i and time t, where $O_{\mathrm{it}}^{s}$ depends on the aggregated (total) number of subjects $N_{\mathrm{it}}^{s}$ at risk in area i at time t. The crude prevalence rate $c_{\mathrm{it}}^{s}$ and expected number of subjects in the population $\left(e_{\mathrm{it}}\right)$ are given as $c_{\mathrm{it}}^{s}=\frac{O_{\mathrm{it}}^{s}}{N_{\mathrm{it}}^{s}}\text{,}$ $e_{\mathrm{it}}=N_{\mathrm{it}}\frac{O_{\mathrm{it}}^{s}}{N_{\mathrm{it}}^{s}}$ . The $O_{\mathrm{it}}$ follows a Poisson distribution with a mean of $\mu_{\mathrm{it}}$ defined as $\left.O_{\mathrm{it}}\right|\omega_{\mathrm{it}}\sim \mathrm{Pois}\left(\mu_{\mathrm{it}}=e_{\mathrm{it}}\omega_{\mathrm{it}}\right)$ . Where, $\log\left(\mu_{\mathrm{it}}\right)=\log\left(e_{\mathrm{it}}\right)+\log\left(\omega_{\mathrm{it}}\right)$ , $e_{\mathrm{it}}$ are the expected cases and $\omega_{\mathrm{it}}$ is a relative risk (RR).

The RR will be decomposed additively into components depending on time, space, and/or both given as:


(1) $$\log\left(\omega_{\mathrm{it}}\right)=\eta_{\mathrm{it}}=\beta_{0}+x_{i}^{T}\beta +v+u+\phi +\left(\gamma +\psi_{i}\right)t$$

where $\beta_{0}$ is the intercept, the overall RR in the district, which is common to all the districts and years, $v={\left(v_{1},\ldots ,v_{s}\right)}^{T}$ is the spatial random effect, $u={\left(u_{1},\ldots ,u_{i}\right)}^{T}$ models spatially-uncorrelated heterogeneity of the outcome variable, $\phi ={\left(\phi_{1},\cdots ,\phi_{T}\right)}^{T}$ is a vector of unstructured temporal effect, γ is the overall time trend (main) effect, and $\psi ={\left(\psi_{1},\ldots ,\psi_{s}\right)}^{T}$ is the area-level time effect (interaction between linear time trend and the district effect). Equation 1 can be expressed in matrix form as


(2) $$\begin{array}{c} \eta =1_{TS}\beta_{0}+X\beta +\left(1_{T}⊗I_{S}\right)v+\left(1_{T}⊗I_{S}\right)u\\ +\left(I_{T}⊗1_{S}\right)\phi +\left(I_{T}⊗1_{S}\right)\gamma +I_{TS}\psi \end{array}$$

where $\eta =\left(\eta_{11},\cdots ,\eta_{T1},\ldots ,\eta_{1S},\ldots ,\eta_{TS}\right)$ , $1_{TS},\;1_{T}$ and $1_{S}$ are columns of ones of length TS,T, and S respectively, $I_{S},I_{T}$ and $I_{TS}$ are S×S,T×T and, TS×TS identity matrices, respectively, and $X=\left(X_{1},\;\cdots ,\;X_{k}\right)$ is the TS×k matrix of the standardized spatiotemporal covariates. The unstructured location level effects were modeled through independent normal distribution, $u_{i}\sim N\left(0,\sigma_{u}^{2}\right)$ . According to the Besag (9), the vector of structured spatial effects $v={\left(v_{1},\ldots ,v_{s}\right)}^{T}$ were assigned by the intrinsic conditional auto-regressive (ICAR), $\left.v_{i}\right|v_{j}\in A_{i}\sim N\left\{\mu_{i},\sigma_{i}^{2}\right\}$ , where $\mu_{i}=\frac{\sum_{j\in A_{i}}v_{j}w_{ij}}{\sum_{j\in A_{i}}w_{ij}}$ and $\sigma_{i}^{2}=\frac{\delta_{v}^{2}}{\sum_{j\in A_{i}}w_{ij}}$ . The spatial dependence parameter for the mean of $v_{i}$ is shown by the weight matrix $\left(w_{ij}\right)$ (21) is stated as:


(3) $$w_{ij}=\{\begin{array}{c} -1\;\mathrm{if}\;\mathrm{locations}\;i\;\mathrm{and}\;j\;\mathrm{are}\;\mathrm{neighors}\;\\ 0\;\;\mathrm{otherwise} \end{array}$$

The vector of structure temporal effect $\gamma ={\left(\gamma_{1},\cdots ,\gamma_{T}\right)}^{T}$ were assigned the first-order random walk (RW1) with prior distribution specified as; $\pi \left(\gamma /\sigma_{\gamma }^{2}\right)\propto \exp\left\{-\frac{1}{2\sigma_{\gamma }^{2}}\left(\gamma^{T}W_{\gamma }\gamma \right)\right\}$ , and $W_{\gamma }$ is the RW1’s structure matrix (32), where the unstructured temporal effect assumes no temporal structure for priors and an independent mean-zero and unknown variance $(\phi_{t}\sim N\left(0,\sigma_{\theta }^{2}\right)$ . Moreover, the vector of interaction effects between location and time $\psi ={\left(\psi_{11},\ldots ,\psi_{S1},\ldots ,\psi_{1T},\ldots ,\psi_{STT}\right)}^{T}$ is assumed to follow a Gaussian kernel distribution with $\pi \left(\psi /\sigma_{\psi }^{2}\right)\propto \exp\left\{-\frac{1}{2\sigma_{\psi }^{2}}\left(\psi^{T}W_{\psi }\psi \right)\right\}$ , where $W_{\psi }=\left(W_{\psi }⊗W_{v}\right)$ .

### Interaction types for spatiotemporal models

**Figure 3:**
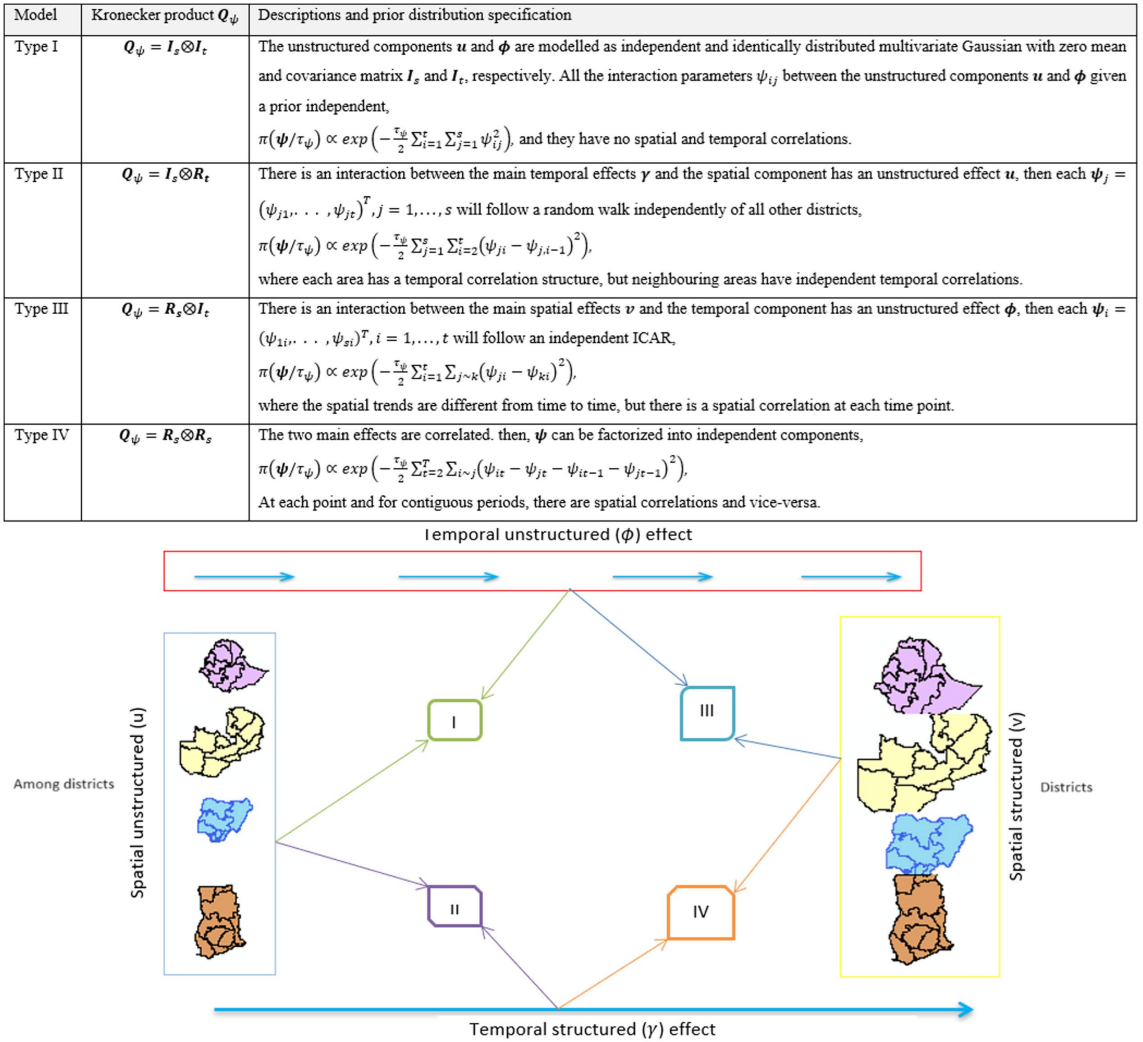
Illustration of the four interaction types.

Four types of spatiotemporal interactions (Equation 2) are (unstructured/structured between location and time); interaction effects are observed (15), and each possible interaction effect is summarized in Figure 3.

In this study, we considered the most complex interaction (type IV), as it incorporates spatial and temporal dependence, which produces spatial and/or temporal confounding (33, 34). Details of the model descriptions are provided in the S1 file. The effect of time T is different for each area because it depends on the covariates in that area, and the spatial effect associated with the $i^{th}$ area is different each time. As a result, this model has time-varying spatial and space-varying temporal effects. Since the disconnected regions can be different in terms of infrastructure, development, demographic, and socioeconomic situations, we assume that the spatial variations in the countries are quite different; hence, we split the random effect of the spatial components into four countries (9, 11, 12, 23). In this study, the U5M rates were aggregated over 37 districts in four sSA countries over four different waves of the DHS datasets. Data from the enumeration areas (EAs) of the districts were also available. To account for the variation in the size of the number of U5M children survey areas, the total number of under-five children (U5C) in the given district was included in the model as an offset (35) variable.

### Model selection criteria

Different techniques were used to select the best model, including the Deviance Information Criterion (DIC) and the Widely Applicable Information Criterion (WAIC), with the lowest values indicating a better fit. For convenience, a DIC value greater than 3 was considered significant (18, 31).

## Results

**Figure 4:**
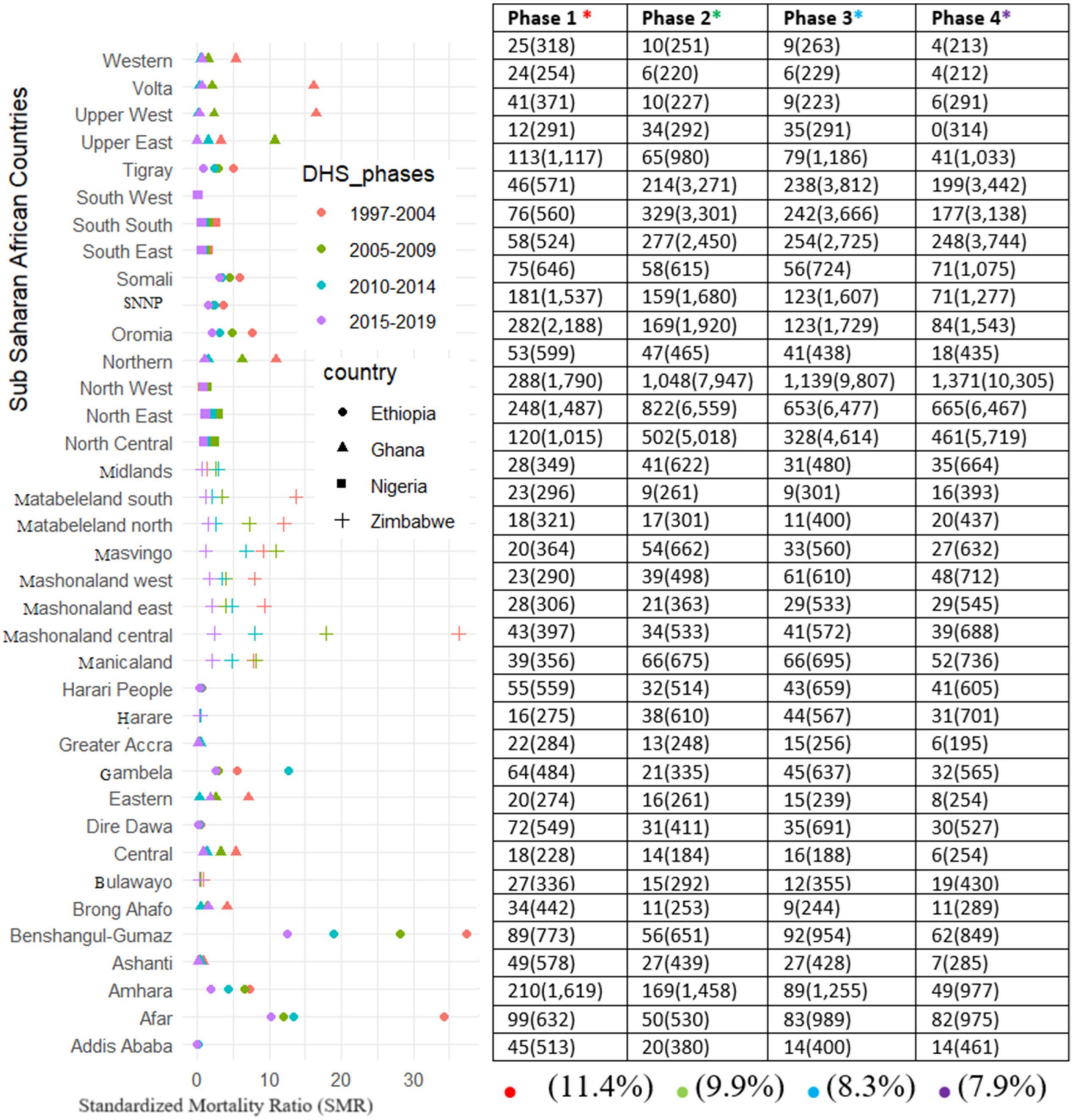
Standardized Mortality Rate and mean under-five mortalities per 1,000 live births and number surveyed (cases) of under-five children for each of the districts across the study period in four sSA countries.

A total of 170,356 under-five children were recorded for all 37 districts for the entire DHS phase (Phase 1: Phase 4) in four disconnected sSA countries (Ethiopia, Ghana, Nigeria, and Zimbabwe). There were 15,467 U5M cases among the children (2,684, 4,544, 4,155, and 4,084 in phase I, II, III, and IV, respectively). For the first phase of the DHS (1997–2004), the districts that had a U5M rate greater than 100 per 1,000 live births included 17 districts (Northeast, Northwest, South–South, and North-central from Nigeria; Afar, Gambela, Oromia, Amhara, Dire Daw, Somali, Tigray, and Harari from Amhara; Upper West from Ghana; and Manicaland in Zimbabwe). However, in the recent DHS phase (2015–2019), almost all districts in Ghana, Addis Ababa, Tigray in Ethiopia, Matabeleland North, Masvingo, and Bulawayo in Zimbabwe had less than 50 U5M rates per 1,000 live births. The prevalence of U5M improved over time (11.4–7.9%) across the study areas. The district pattern in the U5M rate changed considerably throughout the different phases of the DHS data-collection period. Many regions in Nigeria and Ethiopia had the highest mortality among children under-five, but the regions in Ghana had the lowest U5M rate. In the first phase, almost none of the districts achieved any of the goals set to reduce the U5M rates. However, over time, some districts have achieved their goals. Moreover, Figure 4 shows the standardized mortality ratio (SMR) and number of deaths for the years 2000–2020 for the four sSA countries. This result indicates that in the first DHS waves, Benshangul Gumuz and Afar in Ethiopia, Mashonland Central and Matabeleland in Zimbabwe, and Volta and Upper West in Ghana had the highest under-five mortality ratios. Moreover, we observed that SMRs have been decreasing steadily over the last 20 years, indicating and illustrating continuous progress regarding mortality (Figure 4).

The three different models were compared using different statistical metrics (mean deviance, effective number of parameters, and deviance information criteria). The spatiotemporal model (Model 3), with the interaction effects of covariates with both random effects of time and space, had the lowest DIC and WAIC, implying that it was the best-fit model (Table 2). Therefore, the Bayesian inference of fixed-effect parameters with a 95% credible interval for Model 3 was computed using INLA.

**Table 2:** Model comparison and selection for different spatiotemporal models with different specifications for each of the random effects with R-INLA.

Types of models	$\bar{D}$	PD	DIC	WAIC
Model ST1	2613.60	375.14	3412.53	4194.30
Model ST2	305.92	90.34	1133.98	1128.54
Model ST3	303.86	89.37	1131.91	1125.33

PD, effective number of parameters, $\bar{D}$ the mean deviance, ST1-3: spatiotemporal model included in the mathematical Equations 1–3.

**Figure 5:**
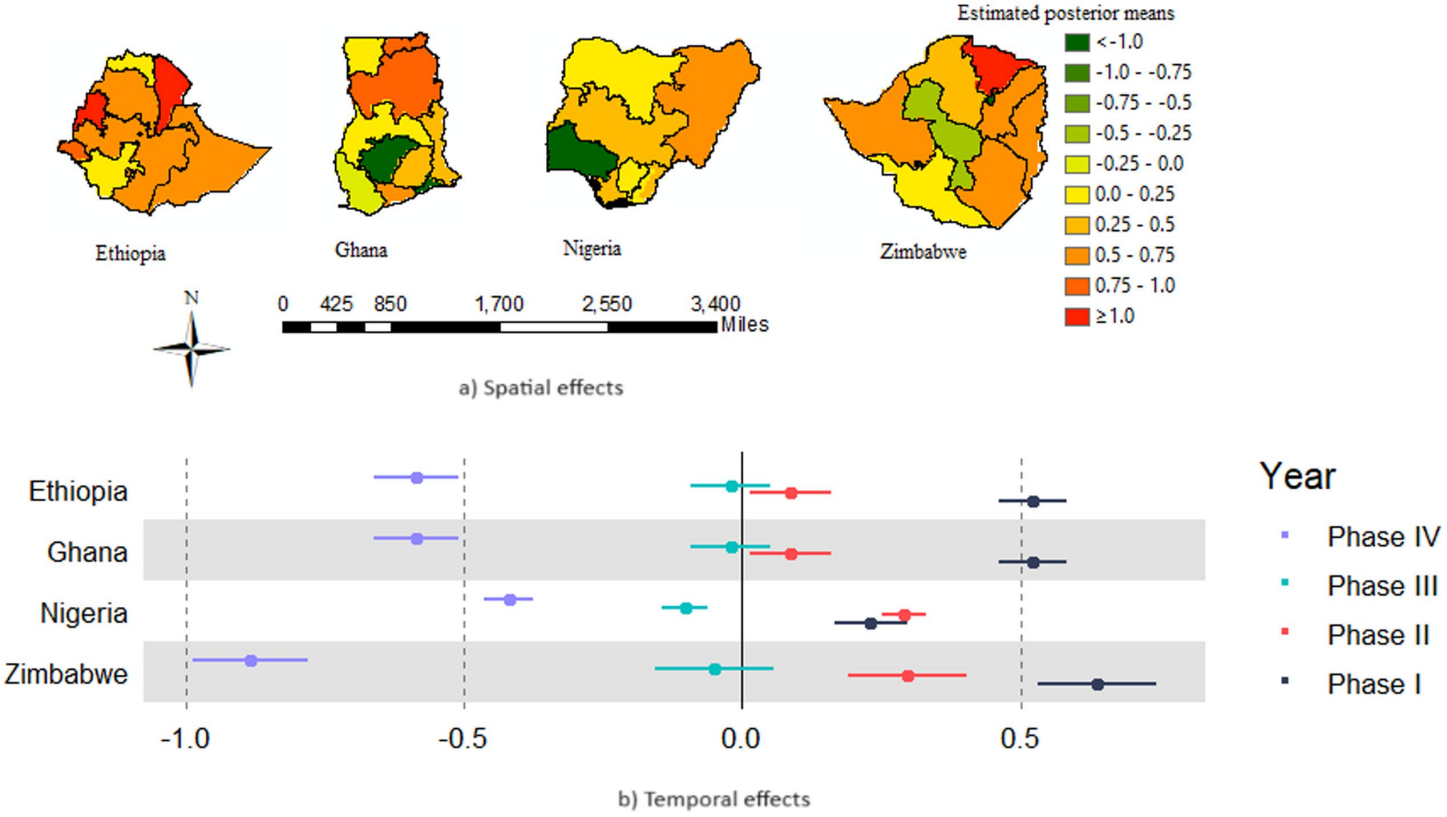
Spatial and temporal effects in the U5M rate across districts of four sSA countries **(A)** mean posterior log odds of U5M for districts **(B)** provides the common posterior temporal mean log odds (trend) of U5M for the districts of each country.

The posterior mean estimates of the marginal spatial log odds for the U5M cases for each of the 37 administrative areas and a summary of the posterior mean estimate of the marginal temporal log odds of the U5M cases are summarized in Figure 5. The upper row shows the posterior log odds that the red color is toward high risk and the green color toward low risk of U5M among the districts across the countries. The spatial map reveals that a high risk is associated with the majority of the districts in Ethiopia and Zimbabwe, whereas a low risk is associated with the majority of the districts in Nigeria and Ghana. Finally, the temporal component revealed the posterior log odds of U5M among the four disconnected countries. The common risk to all districts for each of the disconnected countries and the table of the posterior mean of the main temporal effect in Figure 5 reveal a slight decrease in the global trend over time passes, indicating that there might be an effect on the whole district that produces a decrease in risk of U5M along the phases. The improvement of U5M in Zimbabwe is better than the others, and in Nigeria, it is relatively low, but in all countries, it sharply improved over time.

**Figure 6:**
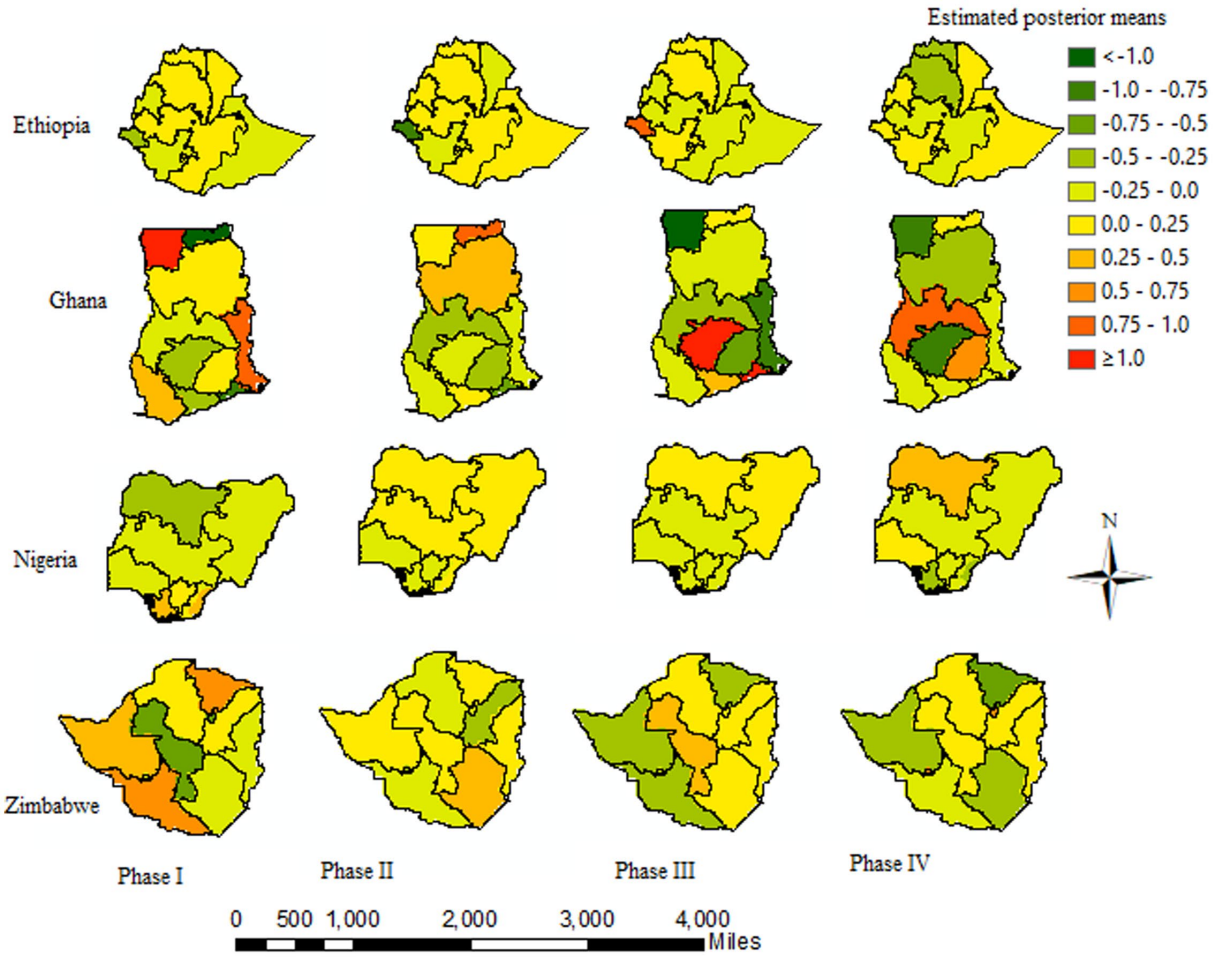
Posterior log odds of U5M by districts over the study period: darker red (high risk) and darker green (lower risk).

The posterior mean estimates of the log odds of the spatiotemporal model for the U5M cases for the 37 districts over the four waves of the DHS datasets are presented in Figure 6. This showed that specific temporal trends vary across districts; hence, including the interaction term in the model is appropriate. The posterior log odds of the spatial effect for a given district were different in each phase of the DHS period. A posterior log odds mean greater than one indicates an excess risk of U5M among children in each of the four sSA countries. Moreover, an increased risk of U5M was observed in the maps that became darker red, and a lower risk became darker green. Over time, from DHS phases 1 to 4, the risk of U5M decreased in the majority of the districts. However, in the majority districts of Nigeria and Ethiopia, the U5M improved slowly, indicating that there are confounding factors associated with the posterior log odds of U5M across a particular district in the country. Most of the districts in Ghana (except Ashanti and Brong Ahafo) were consistently at a lower U5M risk, especially after the third DHS phase. Specifically, for the recent DHS phase (2015–2019), the predicted posterior risk revealed that the highest mortality rates were those in the Somali, SNNP, and Afar regions in Ethiopia; Northwest and Southwest in Nigeria; and Brong Ahafo and Ashanti in Ghana. However, the majority of the regions in Nigeria and Ethiopia have the highest posterior U5M rates compared to Ghana and Zimbabwe (Figure 6).

Table 3 shows the estimated coefficients of covariates (fixed effects) in the selected model, together with the estimated hyperparameters of the spatial, temporal, and spatiotemporal random effects on the under-five mortality cases. Some fixed effects reveal that the 95% credible intervals of the estimates do not contain zero. This suggests that these factors have a major effect on the heterogeneity of the U5M risk across sSA countries. An increase of one unit in the access to improved sanitation index (standard deviation) is associated with a decrease of approximately 34% (exponent of the log odds), 59, 4, and 14% in the relative risk of U5M in Ethiopia, Ghana, Nigeria, and Zimbabwe, respectively. Although an increase of 1 unit index in improved water is associated with 11, 28, 20, and 30% decreases of the relative risk in U5M rate across Ethiopia, Ghana, Nigeria, and Zimbabwe, respectively. Moreover, an increase of one unit index in rural residence settlement and poor wealth quantile index is associated with an increase of around 45, 48, 68, and 15% and 67, 14, 62, and 82%, respectively, in the risk of under-five mortality in Ethiopia, Ghana, Nigeria, and Zimbabwe, respectively. The estimated hyperparameters and proportions of the total variability explained by the spatial, temporal, and spatiotemporal random effects of the spatiotemporal model are summarized in Table 3. From the total variance explained by random effects, the estimated contribution of temporal effects is larger than that of the spatial and interaction effects in all countries.

**Table 3:** Posterior mean, standard deviation, and the 95% credible intervals for the fixed effects for the selected model with R-INLA.

Intercept	sSA countries	Ethiopia	Ghana	Nigeria	Zimbabwe
Variables	Mean (95% Cr.I)	Mean (95% Cr.I)	Mean (95% Cr.I)	Mean (95% Cr.I)	Mean (95% Cr.I)
Intercept	0.41 (0.38, 0.43)	0.54 (0.50, 0.58)	0.39 (0.30, 0.48)	0.09 (0.06, 0.12)	0.57 (0.51, 0.63)
% of children’s nutrition		−0.06 (−0.16, 0.05)	−0.58(−0.73, −0.44)	−0.06 (−0.16, 0.04)	−0.56 (−0.76, 0.35)
% female children		−0.13 (−0.18, −0.08)	0.21 (0.11, 0.31)	−0.02 (−0.06, 0.03)	0.16(0.07, 0.24)
% of children with minimum DDS		0.06 (−0.09, 0.21)	−0.43 (−0.48, 0.41)	−0.37 (−0.48, −0.26)	−0.13 (−0.16, −0.11)
% of child born		0.17 (0.04, 0.31)	0.27 (0.20, 0.35)	0.23 (0.07, 0.38)	−0.44 (−0.62, −0.26)
% of women with literacy		−0.20 (−0.34, −0.07)	−0.11 (−0.14, −0.08)	−0.75 (−0.86, −0.63)	0.14 (−0.04, 0.33)
% of mothers below the median age		0.42 (0.37, 0.48)	−0.35 (−0.51, −0.20)	−0.16 (−0.28, −0.05)	0.56 (0.46, 0.66)
% of women with low autonomy		0.07 (−0.01, 0.15)	−0.51 (−0.86, −0.15)	−0.24 (−0.30, −0.18)	0.04 (−0.12, 0.19)
% of access to safe sanitation		−0.42 (−0.50, −0.33)	−0.90 (−0.97, −0.83)	−0.04 (−0.09, 0.01)	−0.15 (−0.25, −0.05)
% access to safe water		−0.23 (−0.36, −0.11)	−0.33 (−0.51, −0.15)	−0.22 (−0.31, −0.14)	−0.35 (−0.51, −0.18)
% of clear fuels used		−0.35 (−0.51, −0.18)	−0.27 (−0.67, 0.13)	−0.82 (−0.92, −0.73)	−0.44 (−0.58, −0.30)
% of women with media exposure		−0.28 (−0.40, −0.15)	−0.12 (−0.16, −0.09)	−0.44 (−0.55, −0.32)	−0.2 (−0.62, 0.21)
% of the working status of the mother		0.12 (0.07, 0.17)	−0.56 (−0.70, −0.42)	−0.28 (−0.36, −0.20)	0.01 (−0.20, 0.22)
% of the working status of the father		0.16 (0.07, 0.25)	0.47 (0.35, 0.60)	−0.07 (−0.10, −0.03)	−0.1 (−0.20, −0.01)
% of households live in rural areas		0.51 (0.10, 0.91)	0.13 (0.11, 0.16)	0.48 (0.43, 0.54)	0.6 (0.16, 1.04)
% of health facility		−0.31 (−0.48, −0.15)	−0.12 (−0.49,0.24)	−0.41 (−0.52, −0.30)	−0.05 (−17, 0.08)
% of poor wealth quantile		0.37 (0.31, 0.42)	0.39 (0.35, 0.43)	0.52 (0.51, 0.56)	0.14(0.07, 0.22)
Hyperparameters in the model					
Area (spatial effect)	0.57 (0.40, 0.71)	0.63 (0.17, 1.08)	0.581 (0.102, 1.838)	35.9 (0.206, 247.6)	10.5 (0.78, 49.43)
Time (temporal effect)	2.76 (2.10, 3.57)	6.27 (0.11, 39.59)	0.663 (0.001, 4.614)	37.2 (0.361, 246.5)	1.98 (0.02, 12.68)
Area–Time (interaction effects)	1.22 (0.98, 1.54)	5.73 (1.80, 14.07)	0.504 (0.189, 1.117)	14.3 (2.51, 46.63)	3.62 (0.82, 11.15)

The parameters are in the log-odds scale, Cred.I: credible interval.

## Discussion and conclusion

Under-five mortality is a major global public health issue that disproportionately affects children, mainly in less developed nations, including sub-Saharan Africa (36). Previous studies of area-level variation in under-five mortality disparities have been limited to large administrative areas where stable estimates of under-five mortality rates by lower administrative districts can be determined, leaving many sSA countries unexplored. The district-level direct estimates (crude) of certain variables are less common and unstable in showing the real distribution of the variables of interest. The objective of this study was to describe the district-level disparities in U5M rates across the 37 districts using the birth record dataset in four disconnected sSA country files (2000–2019) with the application of spatiotemporal models. A number of models with different assumptions have been used to explore the spatiotemporal effects of covariates in U5M (1, 3, 19–22, 30, 37, 38), but this is the first study to incorporate interactions of spatiotemporal random effects with time-varying confounders to estimate U5M across districts using the R-INLA package for four disconnected sSA countries. The study revealed a significant effect of both time and space main effects on U5M risk, and the effects of either of the two depend on the other and the interaction. Generally, there is notable evidence of a steady decline in the district levels of under-five mortality risk for the 20-year study period, although the levels remain high. These findings are in line with prior studies that reported similar temporal decreases in under-five mortality risk across the sub-Saharan African districts (17, 39–42). However, the U5M across four sSA countries in the period of 2000–2019 reveals a general decline in U5M risks; the mortality rates in the Ethiopia and Nigeria districts were relatively higher than those in Ghana and Zimbabwe. The reason why these districts in Nigeria and Ethiopia are high might be that these two countries are the most populated in Africa. Previous studies have shown the effects of different covariates on the U5M rate using different models (1, 3, 19–21, 39, 40, 43). In the majority of these previous studies, the spatial distribution and variation trends of under-five mortality have been explored at the country level (1, 3, 20, 21, 43), and these studies did not observe the potential interaction effects between the covariates and the random effects of time, space, and their possible interactions. Therefore, our study examined the effects of different covariates on the spatial and temporal trends of under-five mortality rates, as well as spatiotemporal variations across the study areas. Specifically, we found that a child with an undernutrition status is directly associated with, which is in line with the study reported by UNICEF (1, 21). Children living in rural areas are more likely to die than those living in urban areas are. This is in line with the study conducted (1, 3, 17, 20, 21, 35, 37, 41, 43), and this is the fact that individuals living in rural residences are frequently economically worse than their urban counterparts. A child born at a health facility had a lower risk than their home-born counterparts, which is in line with previous studies (21, 22, 39, 42); this is likely because mothers may be provided with an important understanding of health practices needed to improve their nutritional status and that of their families. Moreover, the use of improved health facilities (water and fuel use) is linked to a decrease in the under-five mortality risk because improved water, sanitation, and use of clean fuel will minimize the direct effect of infectious diseases.

Among the given models, the contributions of different time and space components were examined using DIC, and the effective number of parameters was estimated. The best-fitting model captured the temporal dependence structure and spatial autocorrelation of the data and was further improved by incorporating time-varying covariates, accounting for the extra variability that was not captured by the main district and time effects. Most previous studies did not see the potential interaction effects between the covariates and the random effects of time, space, and their possible interactions. One of the aims of the present study was to examine the effects of different covariates on the spatial and temporal trends of under-five mortality rates as well as spatiotemporal variations across the study areas. In our analysis, we addressed the potential challenges associated with spatial confounding caused by the district-level covariates. We also illustrate techniques for the specification of graphs for disconnected regions. We explored whether the spatial patterns of U5M risk differed significantly during the study period and quantified the spatiotemporal interactions across the districts. First, spatiotemporal models incorporate spatial, temporal, and interaction effects without introducing district-level covariates. In the second model, we incorporated neighborhood-level covariates. In our analysis, we addressed the potential challenges associated with spatial confounding accounted for by the district-level covariates. To the best of our knowledge, this is the first study to describe district-level disparities in under-five mortality across sub-Saharan African countries. Describing the district-level variation of under-five mortality rate variation within the country may inform future research on the determinants of these disparities, as well as efforts to reduce inequalities and the burden of under-five mortality risks across the sub-Saharan African countries. This study had some limitations. We specified widely used neighborhood structure definitions: two districts are neighbors if they share a common border (contiguity). However, there are other options, such as distance-based methods that measure the distance between two centroids or points, which are worth further investigation in future research.

## Data Availability

The original contributions presented in the study are included in the article/supplementary material, further inquiries can be directed to the corresponding author.

## Footnotes

^1^ https://dhsprogram.com

^2^ https://www.diva-gis.org/gdata
